# Emergence of colistin resistance in multidrug-resistant *Klebsiella pneumoniae* and *Escherichia coli* strains isolated from cancer patients

**DOI:** 10.1186/s12941-019-0339-4

**Published:** 2019-12-12

**Authors:** Mai M. Zafer, Hadir A. El-Mahallawy, Asmaa Abdulhak, Magdy A. Amin, Mohamed H. Al-Agamy, Hesham H. Radwan

**Affiliations:** 1 Department of Microbiology and Immunology, Faculty of Pharmacy, Ahram Canadian University, 4th Industrial Zone, Banks Complex, 6th of October, Cairo, Egypt; 2 Department of Clinical Pathology, National Cancer Institute, Cairo University, Cairo, Egypt; 3 Department of Microbiology and Immunology, Faculty of Pharmacy, Cairo University, Cairo, Egypt; 4 Department of Pharmaceutics, College of Pharmacy, King Saud University, Riyadh, Saudi Arabia; 5 Department of Microbiology and Immunology, Faculty of Pharmacy, Al-Azhar University, Cairo, Egypt

**Keywords:** Colistin-resistance, *mcr*-*1*, *mcr*-*2*, *mgrB*, *Escherichia coli*, *Klebsiella pneumoniae*, Egypt

## Abstract

**Background:**

Colistin resistance is mainly driven by alterations in the Gram-negative outer membrane lipopolysaccharides and is caused, in most cases, by mutations in *mgrB* gene. However, the recent emergence of plasmid-encoded colistin resistance among *Enterobacteriaceae* strains represents a serious threat to global public health. In this paper we have investigated the rates of colistin resistance and the underlying mechanisms in 450 *Klebsiella pneumoniae* and *Escherichia coli* isolates obtained from cancer patients in Egypt.

**Methods:**

Colistin susceptibility and minimum inhibitory concentrations were determined according to the European Committee on Antimicrobial Susceptibility Testing, by broth microdilution, and by E-test. The *mcr*-*1*, *mcr*-*2* and *mgrB* genes were detected by PCR and then sequenced. Clonal diversity in colistin-resistant *K. pneumoniae* was evaluated by multilocus sequence typing.

**Results:**

Forty (8.8%) colistin-resistant isolates, including 22 *K. pneumoniae* and 18 *E. coli*, were isolated over 18 months. Of these, 50% were carbapenem-resistant, out of which nine were *bla*_*OXA*-*48*_ and seven *bla*_*NDM*-*1*_ positive. The mechanisms of colistin resistance could be revealed only in three of the 40 resistant strains, being represented by mcr-1 in one *bla*_*NDM*-*1*_-positive *E. coli* strain and in one *K. pneumoniae* ST11 and by *mgrB* mutations, detected in one *K. pneumoniae* isolate. None of the studied isolates harbored *mcr*-*2*.

**Conclusions:**

Our results demonstrate a high frequency of colistin resistance in enterobacterial strains isolated from cancer patients, but a low prevalence of the most well known resistance mechanisms.

## Introduction

Antibiotic resistance is one of the most important public health issues worldwide. Severe infections due to multidrug-resistant bacteria, mainly carbapenem-resistant bacteria, in addition to lack of new antibiotics against gram-negative pathogens, have led to a reevaluation of old antibiotics [1]. In this light, colistin has gained clinical value as a last-line drug against serious bacterial infections, since it is effective against nearly all multidrug-resistant gram-negative bacteria. However, a gradual increase in the prevalence of colistin resistance has been noted in the last few years, and elucidation of underlying resistance mechanisms is critical.

Structural modifications of bacterial lipopolysaccharide are the main routes of colistin resistance in gram-negative bacteria. These modifications include addition of 4-amino-4-deoxy-l-arabinose or phosphoethanolamine following chromosomal mutations in genes encoding the two-component systems *PhoPQ* and *PmrAB*, or in *mgrB*, a negative regulator of *PhoPQ* [2]. The phosphoethanolamine transferase *mcr*-*1*, a recently identified horizontally transferable plasmid-mediated colistin resistance gene, is also worrisome, as it has been detected in over 20 countries within 3 months of its identification [2], including in Europe, Asia, South America, North America, and Africa [3–7]. In China, *mcr*-*1* was detected in as many as 20% of animal strains and 1% of human strains [8]. Colistin resistance is most frequently observed in *E. coli*, but is present in various genera, including *Escherichia*, *Klebsiella*, *Salmonella*, *Shigella*, and *Enterobacter* [1]. In Egypt, *mcr*-*1* was first reported in 2016 in an *E. coli* isolate recovered from the sputum of one patient [9]. The *mcr*-*1* gene product adds phosphoethanolamine to the 4′ position of the lipid A moiety of lipopolysaccharides in the outer leaflet of the bacterial outer membrane, significantly reducing the affinity to colistin [10].

Subsequently, Wang et al. [11] described several other MCR homologs (*MCR*-*2*, *MCR*-*3*, *MCR*-*4*, and *MCR*-*5*. Two MCR homologs (MCR-6 and MCR-7) were placed into GenBank, and very recently, the mcr-7.1 gene was found in *K. pneumoniae* of chicken origin in China. *mcr*-*2*, which has about 76.7% nucleotide and 81% amino acid identity to *mcr*-*1*, the archetypal form. Hence, *mcr*-*2* is a similar threat to public health as *mcr*-*1*, although its transfer, origin, and mechanism of resistance are not fully understood [12]. We have now evaluated the rates of colistin resistance in clinical enterobacterial infectious isolates from tertiary Cancer Hospital in Cairo, Egypt, to assess the presence of *mcr*-*1* and *mcr*-*2*, as well as of mutations in *mgrB*.

## Materials and methods

The study was conducted between January 2016 and June 2017 at the National Cancer Institute, Cairo University, Egypt, with approval from the local Ethical Committee. Enterobacterial samples described in this paper are from cultures obtained by the microbiology and clinical pathology department as part of routine care for hospitalized infected cancer patients. No additional clinical specimens were obtained for purposes of research; therefore, informed consent was not required.

### Sample collection

Clinical samples were cultured on blood agar and MacConkey agar (Oxoid Co., England). Isolates were identified by standard microbiological techniques (Colonial morphology, Gram stain, oxidase and the use of several biochemical tests) andVITEK-2 Compact system (bioMerieux, Marcy l’E´toile, France), using *E. coli* ATCC 25922 as control strain. A total of 450 *K. pneumoniae* and *E. coli* strains were recovered.

### Susceptibility testing, detection of ESBL, and determination of minimum inhibitory concentrations

Susceptibility to ampicillin/sulbactam, piperacillin/tazobactam, cefazolin, cefoxitin, ceftazidime, ceftriaxone, cefepim, meropenem, amikacin, gentamicin, tobramycin, ciprofloxacin, levofloxacin, and trimethoprim/sulfamethoxazole was determined by VITEK 2 Compact system. Susceptibility to colistin and tigecycline was evaluated by agar dilution method based on clinical break points defined by the European Committee on Antimicrobial Susceptibility Testing [13]. Minimum inhibitory concentrations for colistin were also measured by broth micro-dilution according to the same standards [13], as well as on E-test strips (bioMérieux, Marcy l’Etoile, France).

### Multilocus sequence typing

Colistin-resistant *K. pneumoniae* isolates were typed by multilocus sequence typing, following the scheme established by the Pasteur Institute (www.pasteur.fr/mlst/Kpneumoniae.html; [14), which is based on the housekeeping genes *gapA*, *infB*, *mdh*, *pgi*, *phoE*, *rpoB*, and *tonB*.

### Characterization of *mcr*-*1* and *mcr*-*2*

Total DNA was extracted by Qiagen DNeasy DNA Extraction Kit (QIAGEN, Crawley, UK) from cultures left at 37 °C overnight in Luria–Bertani (LB) media. Isolates were identified by PCR amplification and sequencing of 16S rRNA, as previously described [15]. Isolates were tested by PCR for plasmid-encoded *mcr*-*1*, using primers CLR5-F (5′-CGGTCAGTCCGTTTGTTC-3′) and CLR5-R (5-CTTGGTCGGTCTGTAGGG-3′), as previously described [8]. Similarly, isolates were tested by PCR for *mcr*-*2*, using *mcr*-*2* full Fw (5′-ATGACATCACATCACTCTTGG-3′) and *mcr*-*2* full Rv (5′-TTACTGGATAAATGCCGCGC-3′) as previously described [16]. Amplified DNA fragments were purified using QIAquick PCR Purification Kit (QIAGEN, Crawley, UK) and sequenced in both directions. Nucleotide and deduced amino acid sequences were analyzed and compared by BLAST, as implemented by the National Center for Biotechnology Information web site (http://blast.ncbi.nlm.nih.gov/Blast.cgi).

### Detection of carbapenem resistance genes

Isolates were screened for the carbapenemases *NDM*, *VIM*, *IMP*, *SIM*, *GIM*, *SPM*, *OXA*-*48*, and *KPC* by multiplex PCR, as previously described [17, 18].

### Analysis of *mgrB*

Using primers *mgrB*-F (5′-AAGGCGTTCATTCTACCACC-3′) and *mgrB*-R (5′-TTAAGAAGGCCGTGCTATCC-3′), *mgrB* was amplified and sequenced in both directions to detect genetic alterations that may drive colistin resistance [19].

## Results

A total of 450 enterobacterial isolates (234 *K. pneumoniae*, 200 *E. coli*, and 16 *Enterobacter*) were collected from hospitalized cancer patients between January 2016 and June 2017, of whom 252 (56%) were males and 198 (44%) were females with age ranges from 1 to < 18 years (pediatrics) (37%), ≤ 55 years (35%) and > 55 years (28%). 440 had received antibiotics 1 month before isolating colistin-resistant Enterobacterial isolate, but none of them had been given colistin. The majority of isolates were from bloodstream infections (n = 263). None of the patients indicated travel within the preceding 12 months. Resistance to cefazolin (414/450; 92%), ceftriaxone (410/450; 91%), ceftazidime (400/450; 89%), cefepime (390/450; 86.5%), trimethoprim/sulfamethoxazole (383/450; 85%), ampicillin/sulbactam (378/450; 84%), levofloxacin (330/450; 73.5%), piperacillin/tazobactam (326/450; 72.5%), ciprofloxacin (321/450; 71.5%), meropenem (240/450; 53.5%), and tobramycin (231/450; 52%) were common, while resistance to gentamycin (202/450; 45%), amikacin (173/450; 38%), and tigecycline (45/450; 10%) was less common (Table 1). As tested by broth microdilution, 40 isolates were resistant to colistin (8.8%), of which 18 (45%) were meropenem-resistant. Similarly, E-tests showed that 36/450 (8%) of isolates were colistin-resistant. Finally, analysis on a VITEK 2 system showed that 140/450 (31%) of isolates produced extended-spectrum beta-lactamase. PCR screening for the most widespread carbapenemases revealed that 16 of the 40 colistin-resistant isolates harbored carbapenemases, with 9/40 positive for *bla*_*OXA*-*48*_ and 7/40 positive for *bla*_*NDM*-*1*_. No other carbapenemases (*KPC*, *VIM*, *IMP*, *SIM*, *GIM*, and *SPM*) were detected.

**Table 1:** Minimum inhibitory concentrations for the studied colistin-resistant isolates

Sample	Isolate	A/S	P/T	CFZ	FOX	CAZ	CTX	FEP	MEM	AMK	GN	TOB	CIP	LEV	TMP/SMX
1	*E. coli*	*8*	*≥* *128*	*≥* *64*	*≥* *64*	*≥* *64*	*≥* *64*	*2*	*8*	≤ 2	*≥* *16*	*≥* *16*	*≥* *4*	*≥* *8*	*≥* *320*
2	*E. coli*	*8*	*≥* *128*	*≥* *64*	*≥* *64*	*≥* *64*	*≥* *64*	*2*	*8*	≤ 2	*≥* *16*	*≥* *16*	*≥* *4*	*≥* *8*	*≥* *320*
3	*E. coli*	*8*	*≥* *128*	*≥* *64*	*16*	*≥* *64*	*≥* *64*	≤ 1	*8*	≤ 2	*≥* *16*	*≥* *16*	*≥* *4*	*≥* *8*	*≥* *320*
4	*K. pneumoniae*	*≥* *32*	*≥* *128*	*≥* *64*	*≥* *64*	*≥* *64*	*≥* *64*	*≥* *64*	*≥* *16*	*≥* *64*	8	*≥* *16*	*≥* *4*	*≥* *8*	*≥* *320*
5	*K. pneumoniae*	*≥* *32*	*≥* *128*	*≥* *64*	*≥* *64*	*16*	*≥* *64*	*≥* *64*	*≥* *16*	*≥* *64*	*≥* *16*	*≥* *16*	*≥* *4*	*≥* *8*	*≥* *320*
6	*K. pneumoniae*	*≥* *32*	*≥* *128*	*≥* *64*	*≥* *64*	*≥* *64*	*≥* *64*	*≥* *64*	*≥* *16*	*≥* *64*	*≥* *16*	*≥* *16*	*≥* *4*	*≥* *8*	*≥* *320*
7	*E. coli*	4	≤ 4	*≥* *64*	8	4	*≥* *64*	*2*	≤ 0.25	≤ 2	*≥* *16*	*8*	*≥* *4*	*≥* *8*	*≥* *320*
8	*K. pneumoniae*	*≥* *32*	*≥* *128*	*≥* *64*	8	*≥* *64*	*≥* *64*	*≥* *64*	≤ 0.25	≤ 2	*≥* *16*	*8*	*5*	*≥* *8*	*≥* *320*
9	*K. pneumoniae*	*≥* *32*	*≥* *128*	*≥* *64*	*≥* *64*	*≥* *64*	*≥* *64*	*≥* *64*	≤ 0.25	4	*≥* *16*	*8*	*≥* *4*	*≥* *8*	*≥* *320*
10	*E. coli*	*≥* *32*	*≥* *128*	*≥* *64*	*≥* *64*	*≥* *64*	*≥* *64*	*≥* *64*	≤ 0.25	≤ 2	*≥* *16*	*≥* *16*	*≥* *4*	*≥* *8*	*≥* *320*
11	*K. pneumoniae*	*≥* *32*	*≥* *128*	*≥* *64*	*≥* *64*	*≥* *64*	*≥* *64*	*≥* *64*	≤ 0.25	*16*	*≥* *16*	*≥* *16*	*≥* *4*	*≥* *8*	*≥* *320*
12	*K. pneumoniae*	*≥* *32*	32	*≥* *64*	8	*16*	*≥* *64*	*16*	≤ 0.25	≤ 2	≤ 1	*≥* *16*	2	1	*≥* *320*
13	*K. pneumoniae*	*≥* *32*	64	*≥* *64*	≤ 4	4	*≥* *64*	*2*	≤ 0.25	4	*≥* *16*	*≥* *16*	2	1	*≥* *320*
14	*K. pneumoniae*	*≥* *32*	*≥* *128*	*≥* *64*	*≥* *64*	4	*≥* *64*	*≥* *64*	*≥* *16*	*≥* *64*	≤1	*≥* *16*	*≥* *4*	*≥* *8*	*≥* *320*
15	*K. pneumoniae*	*≥* *32*	*≥* *128*	*≥* *64*	*≥* *64*	*≥* *64*	*≥* *64*	*≥* *64*	*≥* *16*	*≥* *64*	4	*8*	*≥* *4*	*≥* *8*	*≥* *320*
16	*K. pneumoniae*	*≥* *32*	*≥* *128*	*≥* *64*	8	*16*	*≥* *64*	*2*	*1*	≤ 2	≤ 1	*≥* *16*	*≥* *4*	*≥* *8*	*≥* *320*
17	*K. pneumoniae*	*≥* *32*	*≥* *128*	*≥* *64*	*≥* *64*	*≥* *64*	*≥* *64*	*≥* *64*	*16*	*≥* *64*	*≥* *16*	*≥* *16*	*≥* *4*	*≥* *8*	*≥* *320*
18	*K. pneumoniae*	*≥* *32*	*≥* *128*	*≥* *64*	*≥* *64*	*≥* *64*	*≥* *64*	*≥* *64*	*16*	*≥* *64*	*≥* *16*	*≥* *16*	*≥* *4*	*≥* *8*	*≥* *320*
19	*E. coli*	*≥* *32*	*≥* *128*	*≥* *64*	*32*	*≥* *64*	*≥* *64*	*≥* *64*	*8*	*≥* *64*	2	≤ 1	*≥* *4*	*≥* *8*	*≥* *320*
20	*E. coli*	*≥* *32*	64	*≥* *64*	8	*16*	*≥* *64*	*8*	≤ 0.25	≤ 2	≤ 2	≤ 1	≤ 0.25	1	*≥* *320*
21	*K. pneumoniae*	*8*	≤ 4	≤ 4	≤ 4	≤ 1	≤ 1	≤ 1	≤ 0.25	≤ 2	≤ 2	≤ 1	≤ 0.25	≤ 0.12	*≥* *320*
22	*K. pneumoniae*	*≥* *32*	≤ 4	*≥* *64*	≤ 4	*16*	*≥* *64*	*8*	≤ 0.25	≤ 2	*≥* *16*	4	*≥* *4*	*≥* *8*	*≥* *320*
23	*K. pneumoniae*	4	≤ 4	*≥* *64*	≤ 4	2	*≥* *64*	≤ 1	≤ 0.25	≤ 2	≤ 1	≤ 1	0.5	1	*≥* *320*
24	*K. pneumoniae*	*≥* *32*	*≥* *128*	*≥* *64*	*≥* *64*	*≥* *64*	*≥* *64*	*≥* *64*	*16*	*≥* *64*	*≥* *16*	*≥* *16*	*≥* *4*	*≥* *8*	*≥* *320*
25	*K. pneumoniae*	*≥* *32*	*≥* *128*	*≥* *64*	*≥* *64*	*≥* *64*	*≥* *64*	*≥* *64*	*≥* *16*	*≥* *64*	8	*≥* *16*	*≥* *4*	*≥* *8*	*≥* *320*
26	*E. coli*	*≥* *32*	*≥* *128*	*≥* *64*	*≥* *64*	*≥* *64*	*≥* *64*	*≥* *64*	≤ 0.25	≤ 2	*≥* *16*	*≥* *16*	*≥* *4*	*≥* *8*	*≥* *320*
27	*E. coli*	*≥* *32*	8	*≥* *64*	*16*	*≥* *64*	*≥* *64*	*≥* *64*	≤ 0.25	4	*≥* *16*	*≥* *16*	*≥* *4*	*≥* *8*	*≥* *320*
28	*E. coli*	*≥* *32*	*≥* *128*	*≥* *64*	*≥* *64*	*≥* *64*	*≥* *64*	*≥* *64*	≤ 0.25	≤ 2	*≥* *16*	*≥* *16*	*≥* *4*	*≥* *8*	*≥* *320*
29	*E. coli*	*≥* *32*	*≥* *128*	*≥* *64*	*≥* *64*	*≥* *64*	*≥* *64*	*≥* *64*	*≥* *16*	*≥* *64*	*≥* *16*	*≥* *16*	*≥* *4*	*≥* *8*	*≥* *320*
30	*E. coli*	*≥* *32*	*≥* *128*	*≥* *64*	*≥* *64*	*≥* *64*	*≥* *64*	*≥* *64*	≤ 0.25	*16*	≤ 1	*≥* *16*	*≥* *4*	*≥* *8*	*≥* *320*
31	*K. pneumoniae*	*≥* *32*	*≥* *128*	*≥* *64*	*≥* *64*	*≥* *64*	*≥* *64*	*≥* *64*	≤ 0.25	8	≤ 1	*≥* *16*	*≥* *4*	*≥* *8*	*≥* *320*
32	*E. coli*	*≥* *32*	*≥* *128*	*≥* *64*	*≥* *64*	*≥* *64*	*≥* *64*	*≥* *64*	*≥* *16*	*≥* *64*	*≥* *16*	*≥* *16*	*≥* *4*	*≥* *8*	*≥* *320*
33	*K. pneumoniae*	*16*	≤ 4	8	≤ 4	*16*	≤ 1	≤ 1	≤ 0.25	≤ 2	≤ 1	≤ 1	*≥* *4*	*≥* *8*	*≥* *320*
34	*E. coli*	≤ 2	≤ 4	≤ 4	≤ 4	≤ 1	≤ 1	≤ 1	≤ 0.25	≤ 2	≤ 1	≤ 1	≤ 0.25	≤ 0.12	*≤* *320*
35	*K. pneumoniae*	*≥* *32*	16	*≥* *64*	*≥* *64*	*16*	*≥* *64*	*2*	≤ 0.25	≤ 2	*≥* *16*	*≥* *16*	2	1	*≥* *320*
36	*K. pneumoniae*	*8*	≤ 4	*≥* *64*	≤ 4	*16*	*≥* *64*	*2*	≤ 0.25	≤ 2	≤ 1	≤ 1	*≥* *4*	*≥* *8*	*≥* *320*
37	*E. coli*	*≥* *32*	*≥* *128*	*≥* *64*	*≥* *64*	*≥* *64*	≥ 64	*≥* *64*	*1*	≤ 2	≤ 1	*8*	*≥* *4*	*≥* *8*	*≥* *320*
38	*E. coli*	*≥* *32*	*≥* *128*	*≥* *64*	≤ 4	*16*	*≥* *64*	*8*	≤ 0.25	8	*≥* *16*	*≥* *16*	≤ 0.25	≤ 0.12	*≥* *320*
39	*E. coli*	*≥* *32*	*≥* *128*	*≥* *64*	≤ 4	*16*	*≥* *64*	*8*	≤ 0.25	8	*≥* *16*	*≥* *16*	≤ 0.25	≤ 0.12	*≥* *320*
40	*E. coli*	*≥* *32*	*≥* *128*	*≥* *64*	*≥* *64*	*≥* *64*	*≥* *64*	*≥* *64*	≤ 0.25	≤ 2	*≥* *16*	*≥* *16*	*≥4*	*≥* *8*	*≥* *320*

Minimum inhibitory concentrations were interpreted as resistant (italics) or susceptible (plain text). All of them are µg/mL

*A/S* ampicillin/sulbactam, *P/T* piperacillin/tazobactam, *CFZ* cefazoline, *FOX* cefoxitin, *CAZ* ceftazidime, *CTX* ceftriaxone, *FEP* cefepime, *MEM* meropenem, *AMK* amikacin, *GN* gentamycin, *TOB* tobramycin, *CIP* ciprofloxacin, *LEV* levofloxacin, *TMP-SMX* trimethoprim-sulfamethoxazole

Mutilocus sequence typing of colistin-resistant *K. pneumoniae* revealed seven ST101 strains and three ST383 isolates. Two isolates each of ST147, ST11, ST16, and ST1399 were also detected, along with one isolate each of ST22, ST37, ST785, and ST2193 (Table 2).

**Table 2:** Phenotypic and genotypic characteristics of colistin resistant strains

Isolate number	Isolate	Source	Colistin MIC by broth microdilution (µg/mL)	E-test	ST	OXA-48	NDM-1	mcr-1	mcr-2	mgr-B
1	*E. coli*	Blood	4	0.75		−	+	−	−	
2	*E. coli*	Blood	16	8		−	−	−	−	Unobtainable
3	*E. coli*	Pus	4	3		−	+	+	−	
4	*K. pneumoniae*	Pus	32	8	383	−	−	−	−	
5	*K. pneumoniae*	Blood	4	4	101	−	−	−	−	Unobtainable
6	*K. pneumoniae*	Pus	4	2	16	−	−	−	−	WT
7	*E. coli*	Wound	8	4		+	−	−	−	Unobtainable
8	*K. pneumoniae*	Pus	4	3	11	+	−	−	−	
9	*K. pneumoniae*	Drain	4	2	383	−	−	−	−	
10	*E. coli*	Blood	4	4		−	−	−	−	WT
11	*K. pneumoniae*	Blood	4	3	11	−	−	+	−	WT
12	*K. pneumoniae*	Throat	8	4	101	+	−	−	−	WT
13	*K. pneumoniae*	Blood	4	3	1399	−	−	−	−	WT
14	*K. pneumoniae*	Blood	32	16	147	+	−	−	−	WT
15	*K. pneumoniae*	Blood	4	12	147	−	−	−	−	WT
16	*K. pneumoniae*	Blood	4	4	16	−	−	−	−	
17	*K. pneumoniae*	Nephrostomy	4	3	101	−	+	−	−	
18	*K. pneumoniae*	CVP	16	8	2193	−	+	−	−	WT
19	*E. coli*	Blood	32	8		−	−	−	−	Unobtainable
20	*E. coli*	Pus	8	4		−	−	−	−	Unobtainable
21	*K. pneumoniae*	Blood	32	16	37	−	+	−	−	WT
22	*K. pneumoniae*	Chest tube	32	24	101	−	−	−	−	WT
23	*K. pneumoniae*	Oral	32	16	22	−	+	−	−	WT
24	*K. pneumoniae*	Blood	32	8	383	−	−	−	−	WT
25	*K. pneumoniae*	Blood	4	8	101	−	−	−	−	Unobtainable
26	*E. coli*	CVP	8	2		−	−	−	−	Unobtainable
27	*E. coli*	Blood	32	4		+	−	−	−	
28	*E. coli*	Blood	16	4		+	−	−	−	Unobtainable
29	*E. coli*	Blood	8	4		−	−	−	−	Unobtainable
30	*E. coli*	Blood	16	12		−	−	−	−	
31	*K. pneumoniae*	Blood	16	12	785	−	+	−	−	
32	*E. coli*	Blood	16	8		−	−	−	−	WT
33	*K. pneumoniae*	Blood	32	16	101	−	+	−	−	
34	*E. coli*	Pus	32	8		−	−	−	−	Unobtainable
35	*K. pneumoniae*	Blood	8	4	1399	−	−	−	−	Missense
36	*K. pneumoniae*	Pus	4	4	101	−	−	−	−	
37	*E. coli*	Sputum	8	3		+	−	−	−	
38	*E. coli*	Blood	8	4		+	−	−	−	
39	*E. coli*	Blood	16	4		−	−	−	−	
40	*E. coli*	Blood	16	4		−	−	−	−	Unobtainable

Blank: data was not collected

ST, sequence type; WT, wild type; +, positive result; −, negative result

Genotypic surveys for plasmid-encoded *mcr*-*1* and *mcr*-*2* showed that two of 40 (5%) colistin-resistant isolates harbor *mcr*-*1*, including one of 18 *E. coli* isolates and one of 22 *K. pneumoniae* isolates, which is ST11. These genes were 100% identical to the known *mcr*-*1* sequence (Genbank: NG_050417.1, Liu et al., 2016). The minimum inhibitory concentrations for colistin was 4 mg/L for both isolates. *mcr*-*2* was not detected.

**Fig. 1:**
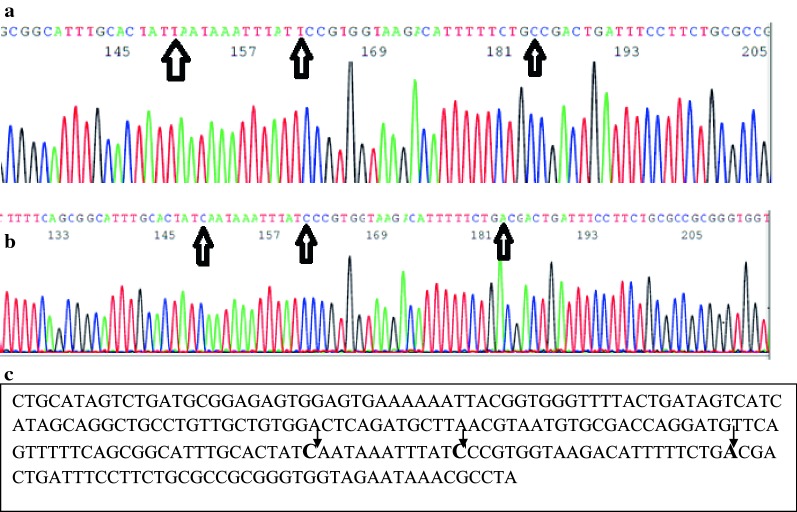
Sequence analysis of *mgrB*. A chromatogram of wild-type (**a**) and mutated *mgrB* (**b**). Isolate 35 harbors a C–A transition that mutates proline to tyrosine at position 178 (**b**). **c** *mgrB* mutations in *K. pneumoniae* isolate 35, with arrows indicating nucleotide changes

Sequencing of the PhoP/PhoQ regulator *mgrB* in 25 select isolates revealed a missense mutation in only one (4%) colistin-resistant *K. pneumoniae*, the genotype of which was ST1399. This mutation (GCC > GAC) mutates proline 178 to tyrosine. Two additional silent mutations were observed with high confidence in this isolate, namely a TAA > CAA mutation at position 144 and a TCC > CCC mutation at position 156. All other isolates harbor wild-type *mgrB* (Fig. 1).

## Discussion

Colistin has become the only viable antimicrobial against aggressive infections due to multidrug-resistant bacteria, and the emergence of plasmid-mediated colistin resistance in Enterobacteriaceae severely compromises its use [10]. Hence, we surveyed colistin resistance rates in multidrug-resistant *K. pneumoniae* and *E. coli* isolated from hospitalized cancer patients at National Cancer Institute, Cairo, Egypt. National Cancer Institute is a tertiary referral hospital, so patients come from different governates in Egypt. The underlying mechanisms driving colistin resistance were investigated by amplification and sequencing of chromosomal *mgrB* and plasmid-encoded *mcr*-*1* and *mcr*-*2*. In the current study, a total of 450 clinical isolates were recovered from cancer patients with hematological malignancies and solid tumors during the study period. More than half of the isolates were obtained from blood stream infections (58%). Gram-negative bacilli causing blood stream infections are frequently detected in cancer patients, and are associated with high mortality. Bacterial bloodstream infections are the leading in case of infectious complications in the course of neutropenia in cancer patients [20]. In line with other studies conducted worldwide, 8.8% of isolates were colistin-resistant, as assessed by broth microdilution. E-test and broth microdilution results were consistent in 36 cases, but the former failed to detect colistin resistance in four isolates, highlighting the reliability of the latter as a reference method for testing colistin susceptibility. Indeed, broth microdilution was found to be the most reliable method for testing colistin susceptibility, as colistin resistance is underestimated by other methods such as agar dilution and disk diffusion. Colistin-resistant *K. pneumoniae* was reported in eastern India [21], and at frequencies of 5.8% and 6.6% in Lao PDR and Thailand, respectively [22]. Similarly, minimum inhibitory concentrations of 3–64 mg/L were reported in 2.4% and 0.7% of isolates in France and Nigeria [22]. *mcr*-*1* was first detected in human isolates in 2011 in Denmark, Germany, Italy, the Netherlands, Spain, Sweden, and the United Kingdom [23]. *mcr*-*1* has since been detected in clinical isolates in Malaysia [7], South Africa [24], Egypt [9], the US [25], and China [26, 27]. Accordingly, this plasmid-encoded gene is of special concern to public health, because it is more easily transmissible than chromosomal colistin resistance genes. In our samples, *mcr*-*1* was present in only two (5%) isolates (*E. coli* and *K. pneumoniae*), implying that colistin resistance is mainly due to chromosomal elements. The minimum inhibitory concentration was 4 µg/mL for both. The *E. coli* isolate, recovered from the site of surgery in a patient, was also carbapenem-resistant, with minimum inhibitory concentration 8 µg/mL. In addition, this isolate harbored the metallo-β-lactamase gene *bla*_*NDM*-*1*_. On the other hand, the *K. pneumoniae* isolate, obtained from a patient with bacteremia, did not harbor carbapenemase genes. Since colistin is not used in the hospital to treat community-acquired infections, this may account for the low prevalence of *mcr*.

*mcr*-*2* was not detected at all, in line with other studies. Indeed, *mcr*-*2* was detected only in Belgium [28], indicating that it is probably dispersed via a different mechanism as *mcr*-*1* [28]. Another possibility is that *mcr* genes are not transmitted from animal and environmental strains to human strains. Additionally, reproducibility of tests for polymyxin/colistin resistance, as well as inconsistencies between assays, as we and others have noted, may hinder detection of isolates with *mcr*-*1* and *mcr*-*2* [29]. Future work will be conducted to screen the other mcr variants (mcr-3, mcr-4 and mcr-5) in infectious enterobacterial isolates recovered from cancer patients.

Cancer patients are frequently subjected to prolonged antibiotic therapy due to neutropaenia. Proven enterobacterial infection requires adminstration of antimicrobial therapy for up to 10 days; this increases the rates of resistance to antimicrobials. High rates of antimicrobial resistance were observed in our samples. For example, 53.5% of isolates were meropenem-resistant. Carbapenem resistance was mediated primarily by *bla*_*OXA*-*48*_ (9/40) and *bla*_*NDM*-*1*_ (7/40), which are the most common carbapenemases in Egypt [30]. Other carbapenemases (KPC, VIM, IMP, SIM, GIM, and SPM) were not detected. In contrast, resistance to tigecycline was rarer (10%), although increasing use of tigecycline to treat life-threatening infections may eventually escalate resistance rates among multidrug-resistant gram negative bacteria. Importantly, the resistance phenotypes of the studied colistin resistant isolates were not identical therefore each isolate was different.

Analysis of *mgrB* in 25 selected colistin-resistant isolates identified 13 with wild-type *mgrB* and one with a missense mutation in *mgrB*. The gene was not detected in the remaining 11 isolates (Table 2). Although the apparently minor role of *mgrB* mutations in colistin resistance among our isolates was unexpected, we note that operons involved in lipopolysaccharide modification are regulated by determinants other than PmrB/MgrB, such as CrrABTCRS [31]. Alternatively, other resistance mechanisms such as accumulation of capsular polysaccharide or efflux pumps may contribute to resistance [32].

Colistin-resistant *K. pneumoniae* isolates in our collection belong to various clones, as assessed by multilocus sequence typing, suggesting considerable genetic diversity present in the same hospital. Of these, ST101 is a major drug-resistant strain, not only in Egypt, but also worldwide [33, 34]. Strikingly, we seem to have found the first mcr-1 positive ST11 strain in Egypt.

## Conclusions

Multidrug resistance is becoming significantly more prevalent in high-risk patients, with the prevalence of colistin resistance increasing at alarming rates in Egypt. Indeed, the plasmid-borne colistin resistance gene *mcr*-*1* was detected in two isolates and is spreading worldwide like plasmid-mediated carbapenemases. Thus, vigilant surveillance of colistin and carbapenem resistance should continue to limit further spread. This is the first report of *mcr*-*1* in *K. pneumoniae* ST11 in Egypt, in a strain recovered from the bloodstream of a hospitalized cancer patient. This is also the first survey for *mcr*-*2* in clinical isolates in the country, although it was not detected. In addition, *mgrB* mutations appear to play only a minor role in driving colistin resistance in this study. The lack of funding is one of the reasons behind the limited investigation of all the possible colistin resistance mechanisms in this study. Thus, chromosomal mutations of the *pmrAB*, and *phoPQ* must be investigated immediately, and further work is needed to fully understand the molecular mechanisms mediating colistin resistance in human enterobacterial isolates.

## Data Availability

All data analysed during this study are included in this published article.

## Abbreviations

EUCAST: European Committee on Antimicrobial Susceptibility Testing
MLST: multi locus sequence typing
ST: sequence type
WT: wild type
